# Endoscopic resection as an independent predictive factor of local control in patients with T1bN0M0 esophageal squamous cell carcinoma treated with chemoradiotherapy: a retrospective study

**DOI:** 10.1186/s13014-021-01972-6

**Published:** 2022-01-20

**Authors:** Tomohiko Miyazaki, Miyako Myojin, Masao Hosokawa, Hidefumi Aoyama, Satoshi Okahara, Hiroaki Takahashi

**Affiliations:** 1 Department of Radiation Oncology, Keiyukai Sapporo Hospital, 1-1 Kita, Hondori-14, Shiroishi-ku, Sapporo, 003-0027 Japan; 2 Department of Surgery, Keiyukai Sapporo Hospital, Sapporo, Japan; 3 Department of Radiation Oncology, Faculty of Medicine, Hokkaido University, North-15 West-7, Kita-ku, Sapporo, 060-8638 Japan; 4 Department of Gastroenterology, Keiyukai Daini Hospital, Hondori-13, Shiroishi-ku, Sapporo, 003-0027 Japan

**Keywords:** Esophageal squamous cell carcinoma, Chemoradiotherapy, Local recurrence, Endoscopic resection, Salvage therapy

## Abstract

**Background:**

Although chemoradiotherapy (CRT) is one of the curative treatments for thoracic esophageal squamous cell carcinoma (ESCC) with submucosal invasion, the risk of local recurrence after CRT remains a clinical problem. This retrospective study aimed to analyze the predictive factors for local recurrence after CRT.

**Methods:**

Ninety-one patients with clinical or pathological (c/p) T1bN0M0 thoracic ESCC who underwent CRT from 2004 to 2017 in our institution were analyzed retrospectively. Sixty-three patients were diagnosed with pathological T1b after undergoing initial endoscopic resection (ER) and treated with additional CRT; meanwhile, 28 patients were clinically diagnosed with T1b and underwent definitive CRT. We investigated the predictors of disease–specific survival (DSS) and local recurrence–free survival (LRFS) by performing univariate and multivariate analyses.

**Results:**

The median observation period was 59.8 months. The 5-year DSS and LRFS rates were 84.3% (95% confidence interval [CI]: 76.1–92.5) and 87.1% (95% CI: 79.1–95.1), respectively. The multivariate analysis revealed no significant predictors associated with DSS. On the contrary, ER (hazard ratio [HR]: 0.11, 95% CI: 0.02–0.48, *p* = 0.003) and tumor length (HR: 6.78, 95% CI: 1.28–36.05, *p* = 0.025) were recognized as independent predictive factors for LRFS. During follow-up, recurrence was observed in 18 patients (19.8%). With regard to the patterns of relapse, local recurrence was the most common in 11 patients, and salvage ER was performed in 9 of 11 patients.

**Conclusions:**

ER and tumor length were independent predictive factors for LRFS. Our study suggested that performance of ER prior to CRT improved the local control in patients with c/p T1bN0M0 ESCC. In addition, most of the patients who experienced local recurrence were treated with salvage ER, which contributed to preserving the organs.

## Introduction

Early stage esophageal squamous cell carcinoma (ESCC) with a depth of cT1a (7th UICC-TMN classification) is usually treated with endoscopic resection (ER). On the contrary, cT1b ESCC with submucosal (SM) invasion is treated with esophagectomy or chemoradiotherapy (CRT) due to the increased risk of lymph node metastasis [1, 2].

A phase II trial (JCOG9708) evaluating the efficacy of definitive CRT (dCRT) for stage I ESCC showed a good long-term outcome with a 4-year overall survival (OS) rate of 80.5% [3]. Following the results of JCOG9708, a randomized controlled trial (JCOG0502) was performed to confirm the non-inferiority of CRT to esophagectomy. Although the randomized part of the trial was not evaluated due to the shortage of cases, in the non-randomized part, the 5-year OS rates were 86.5% for surgery and 85.5% for dCRT (adjusted hazard ratio [HR]: 1.05); this finding further demonstrated the efficacy of CRT [4]. In the JCOG 9708 study, although 87.5% of patients achieved complete remission, the 4-year recurrence-free survival rate was 52.8%. According to existing studies, overall recurrence rate after radiotherapy (RT) or CRT for stage I ESCC was 20–40%, while the intraesophageal recurrence rate was 10–30% [5–9].

ER or esophagectomy can be considered as salvage treatment for local recurrence after CRT, both of which can achieve curative outcomes. Although the impact of salvage treatment for local recurrence on OS is limited, improvement in local control is one of the important issues in CRT from the viewpoint of organ preservation [10, 11]. This retrospective study analyzed the predictive factors related to the long-term prognosis and local control of clinical or pathological (c/p) T1bN0M0 ESCC treated with CRT.

## Methods

### Patients

According to the guidelines for esophageal cancer, patients with cT1a who are diagnosed as pT1b after ER are recommended to be treated according to treatment flow for cT1b, and both the patients with cT1b and pT1b are treated with esophagectomy or CRT. Since the main purpose of this study was to analyze prognostic factors of local recurrence after CRT for ESCC, we included the patients with c/p T1b who were eligible for CRT. In this study, we retrospectively reviewed the medical charts of 91 patients with c/p T1bN0M0 ESCC treated with CRT in our institution from 2004 to 2017, selected according to the following inclusion criteria: (1) aged less than 83 years, which was the age limit of patients eligible for CRT in our institution; (2) with histologically proved squamous cell carcinoma; (3) whose primary tumor site was the thoracic esophagus; (4) whose depth of tumor invasion was diagnosed as c/p T1b; (5) with no clinical evidence of lymph node or distant metastasis; and (6) with no history of chemotherapy and/or RT for esophageal carcinoma.

The evaluation of depth of tumor invasion was based on the Japanese Classification of Esophageal Cancer, 11th edition [12] using esophagogastroduodenoscopy (EGD) and endoscopic ultrasound (EUS) by gastroenterologists. cT1b-SM1 indicates tumor invasion into the upper third of the SM layer, while cT1b-SM2 indicates tumor invasion into the middle third of the SM layer. For cT1a or cT1b-SM1, ER was performed as the initial curative treatment. Patients with pT1b after ER underwent additional CRT to prevent lymph node metastasis. For patients with cT1b-SM2, ER was preceded by diagnostic treatment for tumors that a gastroenterologist determined to be completely resectable, while the rest of the patients were treated with CRT without ER.

### Chemoradiotherapy

RT planning was performed by obtaining three-dimensional computerized axial tomography (CAT) images and using radiation treatment planning systems: Pinnacle versions 8.0–9.10 (Philips, Eindhoven, The Netherlands). The gross tumor volume (GTV) was contoured on the planning CAT images using all available resources. Where necessary, the tumor position was marked endoscopically with clips to determine the GTV. The primary clinical target volume (CTV-P) was identified by adding 2–3 cm margins in the cranio-caudal direction and 0.5 cm margins in the lateral direction from the GTV. In the elective nodal irradiation (ENI) case, the CTV included both CTV-P and all prophylactic regional lymph node areas. In the involved field irradiation (IFI) cases, the CTV was defined as the region including the CTV-P and an optional part of the regional lymph node area. If the GTV was not present after the ER, CTV was considered as equivalent to the prophylactic regional lymph node area. The planning target volume was identified by adding a 1–1.5 cm margins from the CTV.

A different technique was used to perform ENI during the periods of RT. From December 2004 to March 2009, the planned doses of 39.6–65 Gy in 20–26 fractions were delivered to the isocenter with opposed/unopposed portals using the cord-sparing technique. From April 2009 to 2017, a dose of 50.4 Gy in 28 fractions was delivered with anterior/posterior opposed and additional oblique portals [13]. After completing the initial plan, in patients who required radical irradiation, a tumor boost of 9 Gy in 5 fractions was delivered with cord-sparing oblique portals to the primary tumor or tumor bed after ER.

The standard doses of drugs used for chemotherapy were as follows: 700 mg/m^2^ of 5-fluorouracil (5-FU) from day 1 to day 5 and 70 mg/m^2^ cisplatin (CDDP) on day 1. Two courses of 5-FU and CDDP were administered during RT at intervals of at least 3 weeks, depending on the hematological data or the patient’s general condition. In one patient with renal dysfunction, nedaplatin was used instead of CDDP.

### Follow-up

After CRT was completed, all patients were included in the follow-up program. The follow-up started on the first day of CRT and ended on the date of death, date of the patient’s last visit to our hospital, or date that the patient was last known to be alive as confirmed by a telephone interview or a letter from the patient’s referring physician. The EGD and CAT scans were performed every 4–6 months for the first 5 years. When suspected tumor was found by EGD, esophageal biopsies were carried out. Lymph node or distant metastasis was examined by performing a CAT scan or a positron emission tomography/computed tomography scan. Lymph node metastasis was assessed according to the Japanese Classification of Esophageal Cancer, 11th edition [12]. For patients who developed recurrence, salvage treatments were considered, including ER, surgery, chemotherapy, RT, CRT, and other palliative treatments. We calculated the OS rate, disease–specific survival (DSS) rate, and local recurrence-free survival (LRFS) rate from the start date of CRT to the date of the last follow-up. LRFS was defined as recurrence within the esophageal cavity and did not include regional lymph node recurrence and distant metastasis. Toxicities were assessed according to the National Cancer Institute Common Terminology Criteria for Adverse Events version 4.0.

### Statistical methods

The Kaplan–Meier method was used for calculating the survival rates, and the difference in survival curves was compared by the log-rank test. The categorical variables were compared using chi-square test and Fisher’s exact test. For all analyses, a two-sided *p* value of < 0.05 was considered significant. To confirm the predictors of DSS and LRFS, a series of multivariate analyses were carried out using a Cox proportional hazards model. A multivariate analysis was performed for variables with a *p* value less than 0.2 in the univariate analyses. All statistical analyses were conducted using the SPSS software (version 26; IBM SPSS Statistics, Chicago, IL, USA).

## Results

### Patient and tumor characteristics

The patient and tumor characteristics are summarized in Table 1. The median observation period was 59.8 (range, 0.7–190.7) months, and the median age was 69 (range, 47–81) years. Of the 91 patients, 63 were treated with CRT after undergoing the initial ER (ER-CRT group). The remaining 28 patients were treated with dCRT without ER (dCRT group). Three patients failed to complete the treatment due to the occurrence of fever (n = 2) and pneumonitis (n = 1).

**Table 1:** Patient and tumor characteristics in all patients

Characteristic	Number	Percent
Median age (years)	69	(Range 47–81)
Gender		
Male	77	84.6
Female	14	15.4
Performance status		
0	86	94.5
≥ 1	5	5.5
Observation period (month)		
Median	59.8	(Range 0.7–190.7)
Main tumor location		
Upper thoracic	10	11.0
Middle thoracic	54	59.3
Lower thoracic	27	29.7
Tumor length (cm)		
Median	2	(Range 1–9)
Circumference of tumor		
< 3/4	73	80.2
≥ 3/4	18	19.8
Multiple lesion		
No	77	84.6
Yes	14	15.4
Endoscopic resection		
No	28	30.8
Yes	63	69.2
RT field		
IFI	13	14.3
ENI	78	85.7
Radiation dose (Gy)		
< 50 Gy	23	25.6
≥ 50 Gy	68	74.4

*RT* radiotherapy, *IFI* involved field irradiation, *ENI* elective nodal irradiation

Table 2 shows the characteristics and pathological results of 63 patients who underwent ER as the initial treatment. All patients underwent endoscopic submucosal dissection (ESD) with en bloc resection, except for one patient who underwent endoscopic mucosal resection (EMR). Fifty tumors (79.4%) demonstrated pT1b-SM2 invasion. Pathological findings included lymphatic invasion in 20 (31.7%) patients, venous invasion in 14 (22.2%) patients, and incomplete resection in 6 (9.5%) patients.

**Table 2:** Results of endoscopic resection

Characteristic	Number	Percent
Method of ER		
EMR	1	1.6
ESD	62	98.4
Type of resection		
En bloc	62	98.4
Unknown	1	1.6
Pathological tumor depth		
SM1	13	20.6
SM2	50	79.4
Tumor differentiation grade		
Unknown	20	31.7
G1	3	4.8
G2	34	54.0
G3	6	9.5
G4	0	0.0
Lymphatic invasion		
Positive	20	31.7
Negative	38	60.3
Unknown	5	7.9
Venous invasion		
Positive	14	22.2
Negative	45	71.4
Unknown	4	6.3
Horizontal margin		
Positive	6	9.5
Negative or uncertain	57	90.5
Vertical margin		
Positive	1	1.6
Negative or uncertain	62	98.4

*ER* endoscopic resection, *EMR* endoscopic mucosal resection, *ESD* endoscopic submucosal dissection

### Outcomes

The 5-year OS, 5-year DSS, and 5-year LRFS rates were 73.7% (95% CI: 64.1–83.3), 84.3% (95% CI: 76.1–92.5), and 87.1% (95% CI: 79.1–95.1), respectively. During follow-up, 18 patients developed recurrence (Table 3). The most common recurrence patterns were local recurrence in 11 patients (12.1%), followed by regional lymph node metastasis in 6 patients (6.6%), and lung metastasis (1.1%) in 1 patient. As the salvage treatment for local recurrence, ESD was performed in 9 of the 11 patients. Two patients did not undergo salvage treatment due to the coexistence of multiple cancers, and received the best supportive care. Two patients with lymph node metastasis underwent esophagectomy, one patient with lung metastasis was treated with surgical partial lung resection, and the other patient received chemotherapy.

**Table 3:** Summary of recurrence events after CRT

Treatment	RT field	Dose (Gy)	Primary tumor site	Recurrence site	RFS (month)	Salvage treatment
dCRT	ENI	59.4	Lt	Local	7.1	ESD
dCRT	IFI	60	UtMtLt	Local	7.2	ESD
dCRT	ENI	59.4	Mt	Local	8.2	ESD
dCRT	ENI	48.6	Mt	Local	18.9	BSC
dCRT	ENI	59.4	Mt	LN (in field)	26.6	Chemotherapy
dCRT	ENI	59.4	Ut	Local	31.8	BSC
dCRT	ENI	59.4	MtLt	Local	47.5	ESD
dCRT	IFI	65	Mt	LN (in field), Pleura	58.8	Chemotherapy
dCRT	ENI	59.4	LtMt	Local	63.7	ESD
dCRT	ENI	59.4	Lt	Local	76.7	ESD
ER-CRT	ENI	40	Mt	Lung	1.4	Local resection of lung
ER-CRT	ENI	39.6	Mt	LN (out of field)	13.1	Chemotherapy
ER-CRT	ENI	28	Lt	LN (in field)	15.3	Surgery
ER-CRT	ENI	40	Mt	LN (in field)	22.5	Surgery
ER-CRT	ENI	50.4	Mt	Local	29.3	ESD
ER-CRT	ENI	50.4	Mt	LN (in field)	31.0	Chemotherapy
ER-CRT	ENI	39.6	Lt	Local	43.2	ESD
ER-CRT	ENI	50.4	Mt	Local	52.2	ESD

*CRT* chemoradiotherapy, *RT* radiotherapy, *RFS* recurrence-free survival, *dCRT* definitive CRT, *ENI* elective nodal irradiation, *ESD* endoscopic submucosal dissection, *IFI* involved field irradiation, *LN* lymph node, *BSC* best supportive care, *ER-CRT* combined endoscopic resection and CRT

**Fig. 1:**
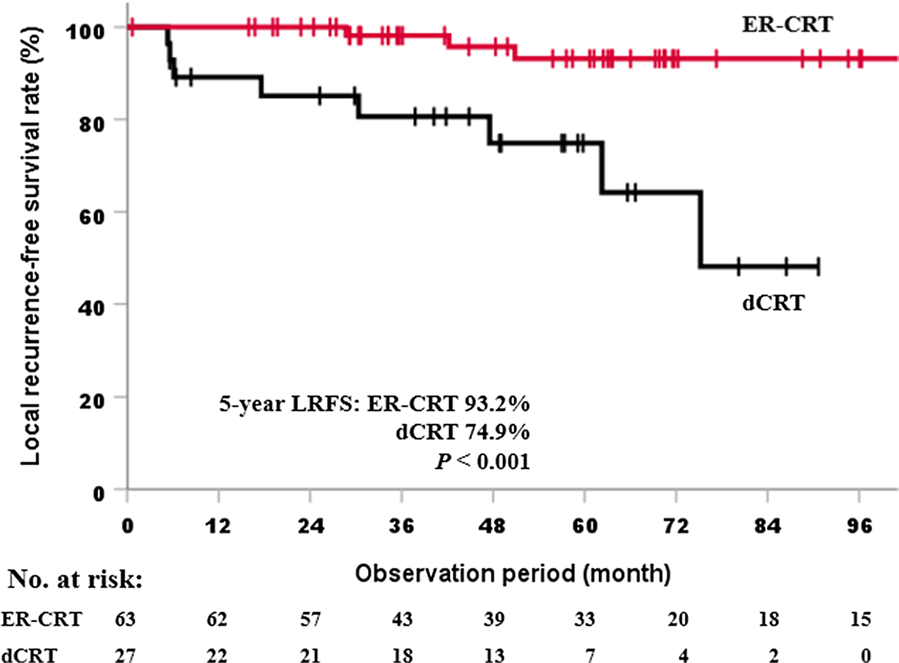
Comparison of local recurrence-free survival rates between the ER-CRT group and the dCRT group

Table 4 shows the 5-year DSS and 5-year LRFS rates for each of the clinical factors as well as; the results of univariate analyses. For the 5-year DSS, the performance status and ER were significant predictive factors (*p* < 0.05); however, the multivariate analysis revealed no significant factors (Table 5). Gender was excluded from the multivariate analysis because all female patients were alive during the observation period. By contrast, the multivariate analysis of the 5-year LRFS showed that tumor length and ER were independent predictive factors, with hazard ratios [HRs] of 6.78 (95% CI: 1.28–36.05, *p* = 0.025) for tumor length and 0.11 (95% CI: 0.02–0.48, *p* = 0.003) for ER (Table 6). The 5-year LRFS rate was 93.2% (95% CI: 85.6–100) in the ER-CRT group, which was significantly higher (*p* < 0.001) than 74.9% (95% CI: 56.9–92.9) in the dCRT group (Fig. 1).

**Table 4:** Univariate analyses for disease-specific survival and local recurrence-free survival rates

Variables	Number	5-year DSS (%)	*p* value	5-year LRFS (%)	*p* value
Age, years					
< 70	43	92.9	0.059	91.8	0.140
≥ 70	48	74.5		82.8	
Gender					
Male	77	81.3	0.094	89.3	0.899
Female	14	100.0		78.8	
Performance status					
0	86	87.3	0.001*	87.0	0.498
≥ 1	5	40.0		100.0	
Tumor location					
Upper thoracic	10	75.0	0.829	77.1	0.567
Middle thoracic	54	85.5		90.9	
Lower thoracic	27	87.4		90.9	
Tumor length					
< 3 cm	46	91.9	0.111	97.4	0.012*
≥ 3 cm	45	76.3		76.7	
Circumference of tumor					
< 3/4	73	85.5	0.681	89.6	0.131
≥ 3/4	18	80.1		79.1	
Multiple lesion					
No	77	85.2	0.757	87.2	0.824
Yes	14	79.6		92.3	
Endoscopic resection					
No	28	72.1	0.045*	74.7	< 0.001*
Yes	63	89.2		93.2	
RT field					
IFI	13	66.7	0.058	92.3	0.649
ENI	78	86.9		87.1	
Radiation dose					
< 50 Gy	23	81.5	0.857	89.1	0.400
≥ 50 Gy	68	85.2		86.3	

*DSS* disease-specific survival, *LRFS* local recurrence-free survival, *RT* radiotherapy, *IFI* involved field irradiation, *ENI* elective nodal irradiation

*p < 0.05

**Table 5:** Multivariate analysis for disease-specific survival rate

Variables	HR (95% CI)	*p* value
Age, years		
< 70	1.0	0.179
≥ 70	2.42 (0.67–8.82)	
Performance status		
0	1.0	0.140
≥ 1	3.06 (0.69–13.58)	
Tumor length		
< 3 cm	1.0	0.337
≥ 3 cm	1.78 (0.50–6.36)	
Endoscopic resection		
No	1.0	0.509
Yes	0.65 (0.18–2.32)	
RT field		
IFI	1.0	0.125
ENI	0.37 (0.10–1.32)	

*HR* hazard ratio, *CI* confidence interval, *RT* radiotherapy, *IFI* involved field irradiation, *ENI* elective nodal irradiation

**Table 6:** Multivariate analysis for local recurrence-free survival rate

Variables	HR (95% CI)	*p* value
Age, years		
< 70	1.0	0.456
≥ 70	1.70 (0.42–6.85)	
Tumor length		
< 3 cm	1.0	0.025*
≥ 3 cm	6.78 (1.28–36.05)	
Circumference of tumor		
< 3/4	1.0	0.441
≥ 3/4	0.57 (0.13–2.40)	
Endoscopic resection		
No	1.0	0.003*
Yes	0.11 (0.02–0.48)	

*HR* hazard ratio, *CI* confidence interval

*p < 0.05

### Comparison of characteristics between the ER-CRT group and the dCRT group

Table 7 summarizes the characteristics of the ER-CRT and the dCRT groups. In the dCRT group, a significant number of tumors exceeded 3/4 of the esophageal circumference (*p* = 0.048). The reason for this is that the guidelines for ER recommend esophagectomy or CRT in patients with tumor that was occupying the entire circumference of the esophagus because of increasing risk of esophageal stricture. Moreover, the total radiation doses were significantly higher in the dCRT group (*p* = 0.001). It was because boost irradiation was required to complete the curative therapy in this group.

**Table 7:** Characteristics of the ER-CRT and the dCRT groups

Characteristic	ER-CRT (n = 63)	dCRT (n = 28)	*p* value
Age, years			
< 70	35	13	0.421
≥ 70	28	15	
Performance status			
0	60	26	0.641
≥ 1	3	2	
Tumor length			
< 3 cm	34	12	0.328
≥ 3 cm	29	16	
Circumference of tumor			
< 3/4	54	19	0.048*
≥ 3/4	9	9	
Multiple lesion			
No	56	21	0.09
Yes	7	7	
RT field			
IFI	7	6	0.194
ENI	56	22	
Radiation dose			
< 50 Gy	22	1	0.001*
≥ 50 Gy	41	27	

*ER-CRT* combined endoscopic resection and chemoradiotherapy, *dCRT* definitive chemoradiotherapy, *RT* radiotherapy, *IFI* involved field irradiation, *ENI* elective nodal irradiation

*p < 0.05

### Toxicities

The adverse events (AEs) are summarized in Table 8. Leukopenia was one of the most common acute AEs, and the incidence of grade 3 or higher leukopenia was 14.3% in our study. Other grade 3 or higher acute AEs included esophagitis (3.3%) and fever (1.1%). Grade 3 or higher late AEs included pleural effusion (1.1%) and pericardial effusion (1.1%). Grade 3 or higher pneumonitis was found in 4.4% of patients, and they were all treated with corticosteroids. One patient died from grade 5 radiation pneumonitis (RP) in this study.

**Table 8:** Adverse events in all patients

	Grade 2	Grade 3	Grade 4	Grade 5
Acute AEs				
Leukopenia	21 (23.1)	10 (11.0)	3 (3.3)	0 (0.0)
Dermatitis	5 (5.5)	0 (0.0)	0 (0.0)	0 (0.0)
Esophagitis	10 (11.0)	3 (3.3)	0 (0.0)	0 (0.0)
Nausea	4 (4.4)	0 (0.0)	0 (0.0)	0 (0.0)
Pneumonitis	0 (0.0)	1 (1.1)	0 (0.0)	0 (0.0)
Fever	3 (3.3)	1 (1.1)	0 (0.0)	0 (0.0)
Late AEs				
Esophageal stenosis	6 (6.6)	0 (0.0)	0 (0.0)	0 (0.0)
Pneumonitis	1 (1.1)	1 (1.1)	1 (1.1)	1 (1.1)
Pleural effusion	1 (1.1)	1 (1.1)	0 (0.0)	0 (0.0)
Pericardial effusion	6 (6.6)	1 (1.1)	0 (0.0)	0 (0.0)

AEs were evaluated according to the Common Terminology Criteria for Adverse Events version 4.0

*AEs* adverse events

## Discussion

In this retrospective study of c/p T1b ESCC, the multivariate analysis did not reveal any statistically significant predictors of DSS; in contrast, tumor length (*p* = 0.025) and ER (*p* = 0.003) were recognized as independent predictors of LRFS. A previous study reported tumor length as a predictor of local control. Ishikawa et al. analyzed patients treated with RT alone for stage I ESCC and reported that local recurrence tended to be more common in those with tumor larger than 5 cm [14]. We did not find any studies conducted in the CRT group that indicated tumor length as a risk factor for local recurrence. Most of the studies adopted a median value as the threshold of tumor length. Although it is difficult to set an absolute standard value because a median value of the tumor length varies among studies, more careful follow-up is needed for the tumor of larger size.

Meanwhile, several studies have shown that a combination of ER and CRT can improve local control. Yoshimizu et al. compared the long-term results of the ER-CRT group and the dCRT group. Although no significant difference was observed in the 5-year OS between the two groups, the ER-CRT group showed significantly higher local control rate (*p* < 0.05) [15]. Kawaguchi et al. reported that 19.4% of patients in the dCRT group had local recurrence, while none of the patients in the ER-CRT group had local recurrence [16]. Hamada et al. also reported that local recurrence occurred in 3% (2/66) of the patients in the ER-CRT group, which was lower than that in the conventional dCRT group [17]. In our study, we confirmed that ER is a significant predictor of local control using the multivariate analysis. This result reinforces the validity of existing reports. With regard to the efficacy of combined ER and CRT treatment for stage I ESCC, Minashi et al. conducted a prospective study (JCOG0508) and reported a 3-year OS rate of 90.7% in the ER-CRT group [18]. In the study, cT1b-SM2 tumor, which was not an indication for ER according to the previous guidelines, was included, and therapeutic diagnostic ER was performed in 35.6% of the patients with cT1b-SM2 tumor. The results of the JCOG0508 study suggested that the combination of ER and CRT can be considered as a useful treatment even for cT1b-SM2 tumor, for which complete resection by ESD is expected. In our study, 79.4% of the patients who underwent ER had pT1b-SM2 tumor and showed favorable long-term results. In addition, as we confirmed in this study, ER is a predictor of improved local control, which may reduce the number of patients requiring salvage surgery due to local recurrence. A combination of ER and CRT treatment allows organ preservation for stage I ESCC and is considered a promising treatment strategy.

In 2003, Hattori et al. reported the results of the first EMR performed as a salvage treatment for local recurrence after CRT in Japan [19]. Consequently, several studies have been conducted to examine the effectiveness and safety of salvage ER after CRT [20–22], it has been regarded as important to detect recurrence after CRT while tumor remains in the superficial layer. In our institution, patients treated with CRT underwent follow-up EGD and CAT scans every 4–6 months during the first 5 years after CRT. In this study, local recurrence was observed in 11 patients during follow-up, and salvage ER was performed in 9 of the 11 patients. This result suggests that patients treated with CRT should be monitored for any signs of local recurrence, and efforts should be made for the early detection of local recurrence by performing endoscopic observations at an appropriate frequency.

Grade 3 or higher pneumonitis was found in 4.4% of patients. This value is slightly higher than that reported in previous studies (1–3%) [4, 8, 18]. We have previously reported the relationship between respiratory toxicities and the percentage volume of the whole lung receiving at least 5–40 Gy (V5–V40) in patients with thoracic ESCC treated with ENI [13]. In the study, V5 and V10 in all lungs were significantly higher in the group with grade 2 or higher RP. V5 (< 55%), V10 (< 37%) and V20 (< 25%) were used as dose constraints; ENI therapy was safely implemented by reducing the risk of adverse events. There was no significant difference in grade 3 or higher AEs between the ER-CRT group and the dCRT group. Esophageal stricture is known to be a problem as a late AEs after ER. In this study, out of 63 patients who underwent ER, grade 2 esophageal stricture was found in 6 patients (9.5%), but no grade 3 or higher was found. Minashi et al. also reported that grade 3 esophageal stricture was observed in one patient (0.6%) after ER, however no other severe AEs were observed and the safety of ER-CRT was acceptable [18].

In 2016, we reported that PS was an independent prognostic factor for DSS and disease-free survival for patients with T1N0M0 ESCC [23]. In the study, cT1a tumors were also included and 33.8% of the patients were treated with RT alone. Since CRT is recommended in the Japanese guidelines [1], we thought it necessary to limit the analysis to patients treated with CRT. In the present study, we retrospectively analyzed the factors associated with local recurrence after CRT for ESCC, and included both the patients with pT1b who underwent ER and the patients with cT1b who did not undergo ER. The multivariate analysis showed that ER was one of the predictors of local recurrence, and the diagnostic accuracy of cT1b in the dCRT group was a problem in interpreting the results. A clinical depth of tumor invasion was diagnosed by EUS along with the findings of magnifying endoscopy. According to the meta-analysis on EUS, the sensitivity and specificity of cT1b diagnosis of superficial esophageal cancer by EUS were both 86% (diagnostic accuracy: 86%), and the area under the curve calculated from the summary receiver operating characteristic curve was reported to be 0.93 [24]. At our institution, a diagnostic accuracy of cT1b ESCC by EUS is about 90%. In addition, the Japanese guidelines recommends that EUS should be used to evaluate the clinical depth of tumor invasion. Based on the above, the possibility of cT1a and cT2 tumors being mixed in the dCRT group was low, and we considered it was reasonable to include the patients with pT1b and cT1b in this study. However, this point was also the limitation of the present study. In the review of adjuvant therapy after ER, Tsou et al. mentioned that there were differences in clinical and pathological diagnosis between patients treated with or without ER, which should be kept in mind when interpreting the results [25]. Our study suggested the benefit of ER prior to CRT even in cT1b tumor, and we hope that our findings will help to conduct a prospective trial comparing the outcomes of ER-CRT and dCRT groups in patients with cT1b tumor.

This study has several other limitations. A nonrandomized and retrospective analysis was performed using the data from a single institution. Other studies comparing the ER-CRT and dCRT groups also mentioned the presence of selective bias; therefore, we attempted to reduce the effects of confounding factors by performing a multivariate analysis. Notably, we were able to reinforce the validity of the results of existing studies by confirming that ER was a predictor of local control even in the multivariate analysis.

## Conclusions

This study confirmed that tumor length and ER were predictors of local recurrence after CRT in the multivariate analysis. ER was also useful as a salvage treatment for local recurrence, and can be expected to improve the organ preservation rate.

## Data Availability

The datasets used and/or analyzed during the current study are available from the corresponding author on reasonable request.

## Abbreviations

5-FU: 5-Fluorouracil
AEs: Adverse events
CAT: Computerized axial tomography
CDDP: Cisplatin
CI: Confidence interval
c/p: Clinical or pathological
CRT: Chemoradiotherapy
CTV-P: Primary clinical target volume
dCRT: Definitive chemoradiotherapy
DSS: Disease-specific survival
EGD: Esophagogastroduodenoscopy
EMR: Endoscopic mucosal resection
ENI: Elective nodal irradiation
ER: Endoscopic resection
ESCC: Esophageal squamous cell carcinoma
ESD: Endoscopic submucosal dissection
EUS: Endoscopic ultrasound
GTV: Gross tumor volume
HR: Hazard ratio
IFI: Involved field irradiation
LRFS: Local recurrence-free survival
OS: Overall survival
RP: Radiation pneumonitis
RT: Radiotherapy
SM: Submucosal
